# Effects of feeding a negative dietary cation and anion difference diet to twin-bearing Merino ewes in late gestation on parturition outcomes

**DOI:** 10.1093/jas/skae266

**Published:** 2024-09-12

**Authors:** Amy L Munn, William H E J van Wettere, Alyce M Swinbourne, Ian J Lean, Alice C Weaver

**Affiliations:** Davies Livestock Research Centre, The University of Adelaide, Roseworthy, South Australia 5371, Australia; Davies Livestock Research Centre, The University of Adelaide, Roseworthy, South Australia 5371, Australia; Turretfield Research Centre, South Australian Research and Development Institute, Rosedale, South Australia 5350, Australia; Scibus, Camden, New South Wales 2570, Australia; Turretfield Research Centre, South Australian Research and Development Institute, Rosedale, South Australia 5350, Australia

**Keywords:** calcium, lamb survival, late gestation, magnesium, maternal supplementation, sheep

## Abstract

In Australia, dystocia is responsible for 53% of lamb mortalities, and calcium deficiencies may be a contributing factor. A negative dietary cation–anion difference (**DCAD**) diet can increase calcium concentrations in sheep. Therefore, this study aimed to investigate the effects of a negative DCAD diet on metabolic state, mineral status, and parturition duration in ewes compared with those fed a positive DCAD diet. At approximately day 130 of gestation (**dG**), 71 twin-bearing ewes were placed in the following treatment groups; ewes receiving a positive DCAD TMR (total mixed ration; DCAD of total diet = 281.8 mEq/kg DM; *n *= 35) and twin-bearing ewes receiving a negative DCAD TMR (DCAD of total diet = −89.0 mEq/kg DM; *n *= 36). Urine and blood were sampled on dG 130, 140, and 145, and blood was also sampled at the onset of parturition and 4 h postpartum. Urine was analyzed for pH and blood was analyzed for metabolites, mineral concentration, and acid–base balance. Lambs’ liveweight, rectal temperature, blood glucose and lactate, and body morphology were measured. Serum phosphate concentrations at dG 145 were significantly lower for negative DCAD ewes compared with positive DCAD ewes (1.9 ± 0.1 vs. 2.1 ± 0.1 mmol/L, *P *= 0.047). Ionized calcium (*P *= 0.09) and serum magnesium (*P* = 0.09) prepartum were marginally greater in the negative DCAD ewes (1.35 ± 0.06 and 1.06 ± 0.03 mmol/L, respectively) compared with the positive DCAD ewes (1.18 ± 0.08 and 0.98 ± 0.04 mmol/L, respectively). Urine pH was lower in the negative DCAD ewes compared with positive DCAD ewes at both dG 140 (7.38 ± 0.17 vs. and 8.10 ± 0.19. *P *= 0.01) and dG 145 (and 7.20 ± 0.19 vs. 8.25. *P *< 0.01). The birth interval between the first the second-born lamb was shorter in the negative DCAD ewes compared with the positive DCAD ewes (*P *= 0.02), but no differences in lamb survival or lamb viability (*P *> 0.05) were seen. The negative DCAD diet reduced parturition duration, most likely due to the marginally greater ionized calcium and magnesium concentrations. Despite this improvement, the negative DCAD ewes did not reach urinary acidification, indicating that the marginally significant greater ionized calcium and serum magnesium concentrations were due to the magnesium in the diets and not metabolic acidosis. Further research testing a negative DCAD diet that can achieve the target urine pH is required to determine whether this diet can decrease parturition duration and improve lamb viability.

## Teaser

This study found that a negative DCAD diet fed to twin-bearing Merino ewes during late gestation reduced the birth interval between the first and second-born lamb.

## Introduction

In Australia, approximately 20% of lambs die prior to weaning (Hinch and Brien, 2014), 53% of which can be attributed to dystocia (prolonged and/or difficult birth), which includes stillbirths and deaths from oxygen deprivation soon after birth (Bruce et al., 2021). Furthermore, approximately 35% of ewe mortalities are attributable to dystocia. These ewe and lamb mortalities associated with dystocia cost the industry approximately $780 million AUD annually, making it a significant economic problem for Australian sheep producers as well as a substantial animal welfare concern (Bruce et al., 2021).

There are many factors that cause dystocia, such as fetal number and lamb birth weight, as well as ewe liveweight, parity, age, body condition score (**BCS**), nutrition, and genetics (Dwyer and Lawrence, 2005; Dwyer and Bünger, 2012; Refshauge et al., 2016; Horton et al., 2017; Jacobson et al., 2020). Nutrition, particularly an inadequate supply of energy and minerals has been linked to dystocia; however, there are limited data describing how this affects the parturition process in sheep (Friend et al., 2020; Jacobson et al., 2020).

An imbalanced supply of minerals is commonly found in modern pastures grazed around parturition (Masters, 2018). The study by Hocking Edwards et al. (2018) reported that a significant number of sheep grazing lush pastures in southern Australia may be at risk of subclinical hypocalcemia and hypomagnesemia in the winter. Furthermore, calcium and magnesium are also lacking in vegetative cereal crops such as wheat, barley, and oats (Masters et al., 2018a). The dietary cation and anion difference (**DCAD**) of pasture may contribute to these deficiencies. The DCAD refers to the difference in cations (sodium and potassium) and anions (chloride and sulfur) in feed (calculated by [Na + K] − [Cl + S] in milliequivalents [mEq]). Pastures that are positive in DCAD cause a shift in the acid–base balance of the ewe which may lead to metabolic alkalosis, impairing the regulation of calcium and predisposing the ewe to a calcium deficiency (Masters et al., 2018b; Friend et al., 2020).

In dairy cows, hypocalcemia and subclinical hypocalcemia are known to increase the risk of dystocia by causing weak uterine contractions during parturition whereas this relationship is less clear in sheep (Goff, 2008; Hossein-Zadeh and Ardalan, 2011). A common preventative measure for hypocalcemia is to feed dairy cows an acidogenic diet to shift the acid–base balance of the animal to mild acidosis (Goff, 2008). Mild acidosis stimulates parathyroid hormone secretion (an important hormone in calcium homeostasis), which upregulates calcium mobilization from bone and mitigates hypocalcemia (Vagg and Payne, 1970; Horst et al., 1997; Roche et al., 2003; Goff, 2008). Feeding a negative DCAD supplement (containing magnesium chloride, calcium sulfate, and salt, in the ratio 12.5:32.5:55.0) improved circulating calcium and magnesium concentrations in ewes grazing cereal crops; however, the effects on parturition duration and lamb survival were not reported (Masters et al., 2018b). In a recent study, with the same supplement mentioned above, in the form of a loose lick, provided to ewes during late gestation grazing pasture did not improve lamb survival; however, the sheep did not consume the targeted level of supplement (Robertson et al., 2022).

In southern Australia, a significant number of grazing ewes are at risk of subclinical calcium and magnesium deficiencies, therefore more research is required to determine the effects of this on parturition outcomes, with an overall aim to reduce ewe and lamb mortalities. Therefore, the aim of this study was to compare the effects of feeding ewes a negative or a positive DCAD diet on blood metabolites, mineral status, and parturition duration in an indoor controlled setting. It is hypothesized that ewes consuming a negative DCAD diet during late gestation have greater circulating calcium and magnesium concentrations and in turn, have a shorter parturition duration and greater lamb viability compared with ewes fed a positive DCAD diet.

## Materials and Methods

This experiment was approved by the Primary Industries and Regions South Australia Animal Ethics Committee (#07/22) and conducted in accordance with the “Australian code for the care and use of animals for scientific purposes 8th edition” (National Health and Medical Research Council, 2013). All animal work was conducted at the South Australian Research and Development Institute, Turretfield Research Centre, Rosedale SA (−34.552°S, 138.832°E).

### Animal selection and housing

Merino ewes were naturally mated and rams were with ewes for approximately 4 wk. On approximately 69 d of gestation (**dG**), ewes were pregnancy scanned to determine fetal number and fetal size, after which 71 twin-bearing ewes were selected for the trial. On approximately dG 130, the ewes moved to indoor, individual lambing pens (2.4 m^2^) and allocated to receive either a positive DCAD diet (positive DCAD; *n *= 35), or a negative DCAD diet (negative DCAD; *n *= 36) based on liveweight (**LW**) and BCS. The trial was conducted in 2 replicates based on fetal size at pregnancy scanning to differentiate early (replicate 1; *n *= 47) and late (replicate 2; *n* = 24) mated ewes. Ewes received a total mixed ration (**TMR**) diet (1.35 kg/d AF; Tables 1 and 2) containing lupins, barley, oats, and a mineral premix fed twice daily (morning and afternoon). The TMR of both diet groups received an acidogenic protein meal Bio-Chlor (Arm & Hammer Animal Nutrition, Princeton, NJ). Bio-Chlor was used as a palatable acidogenic protein meal to acidify the diet and to maintain iso-nitrogenous diets. Sodium bicarbonate was added to the positive DCAD TMR to ensure the positive DCAD TMR had a positive DCAD similar to pastures in southern Australia (ranging from 0 to 760 mEq/kg DM; Roche et al., 2000; Hocking Edwards et al., 2018; Robertson et al., 2022). Both treatment groups were given additional access to 200 g of oaten chaff or oaten hay (separate to the TMR) and had ad libitum access to fresh drinking water. The addition of oaten hay brought the estimated DCAD of the positive DCAD group to +281.8 mEq/kg per kg DM and the estimated DCAD of the negative DCAD diet to −89.0 mEq/kg DM. Any feed residuals were recorded each morning. Dietary treatments continued until 3 d postpartum. Ewes were weighed and body condition was scored weekly to ensure healthy weights were maintained during late gestation.

**Table 1.** Composition of the positive and negative TMR fed during the trial (kg/t)

Feed ingredient, kg/t AF	Positive TMR	Negative TMR
Ground barley	258.7	263.4
Whole oats	295.6	301.0
Lupins	332.6	338.6
Sugar sucrose	7.4	9.0
Mineral premix	1.5	1.5
Dolomitic limestone	59.1	45.1
BioChlor^1^	22.2	22.6
Magnesium sulfate	—	7.5
Calcium sulfate	—	11.3
Sodium bicarbonate	22.2	—
Magnesium oxide	0.7	—

^1^Acidogenic protein meal (Bio-Chlor, Arm & Hammer Animal Nutrition).

Abbreviation: TMR, total mixed ration.

**Table 2.** Diet specifications for each feed component (FeedTest, Agrifood Technology, Victoria, Australia)

	Oaten hay	Oaten chaff	Positive TMR	Negative TMR
DM, %	90.1	88.1	92.5	91.3
Ash, % DM	6.9	6.2	4.6	3.8
Fat, % DM	3.5	4.0	7.3	5.8
CP, % DM	6.1	5.4	18.3	18.3
ADF, %DM	35.2	31.3	11.3	12.5
NDF, % DM	62.2	58.1	26.3	26.3
ME, MJ/kg DM^1^	7.4	8.1	14.0	13.3
Calcium, g/kg	1.8	1.4	24.0	14.0
Potassium, g/kg DM	10	9.7	5.1	4.9
Magnesium, g/kg DM	1.2	1.0	1.9	2.0
Sodium, g/kg DM	8.3	5.5	7.7	0.64
Phosphorus, g/kg DM	1.2	1.4	2.8	2.7
Sulphur, g/kg DM	0.78	0.78	2.0	3.4
Chloride, % DM	1.15	1.17	0.17	0.24
DCAD, meq/kg DM^2^	242	111	287	−125

^1^ME was calculated by using the following equation: ME = 0.858 + (0.138 × dry organic matter digestibility %) + (0.272 × fat %).

^2^DCAD was calculated by using the following equation: DCAD = (Na + K) − (Cl + S).

Abbreviations: DCAD, dietary cation and anion difference; TMR, total mixed ration.

From dG 147 ewes were monitored 24 h/d for the onset of parturition through both direct observation and video surveillance. Straw bedding was provided to ewes when signs of parturition were shown. Seventy-two hours postpartum, ewes and their lamb/s were moved and monitored outdoors if the lamb/s were viable. Approximately 10 d after the last lamb was born ewes and lambs were returned to a paddock and monitored weekly until lambs were approximately 39 d of age.

#### Ewe prepartum measures

Two hours after feeding on dG 130 (baseline), 140, and 145, a 10-mL blood and approximately 30 mL urine sample were collected from ewes. The blood samples were collected via jugular venepuncture using a 10-mL syringe and 18 G 1.5″ needle, then dispensed evenly across a 9-mL lithium heparin vacutainer tube and 9-mL clot activator vacutainer tube (BD Vacutainer, BD, Belliver Industrial Estate, Plymouth, UK) and stored on ice upon collection. Blood ketones, glucose (Abbots Freestyle Optium Neo. Melbourne, VIC, Australia), blood pH, and ionized calcium (LAQUAtwin pH-22 compact pH meter and LAQUAtwin Ca-11C bovine calcium meter, Horiba Advanced Techno Co., Ltd, Kyoto, Japan) were measured with the remaining blood in the syringe. Blood samples in lithium heparin tubes were processed within 1 h of collection by centrifuging at 3,024 × *g* for 15 min. The blood samples in clot activator tubes were stored at 4 °C for 24 h to allow the sample to clot before being centrifuged as above. The plasma and serum were extracted and divided into 2 aliquots stored at −80 °C. The urine samples were collected opportunistically by releasing ewes from their pens and following behind until urination occurred, then the urine was transferred into a 70-mL plastic pot (Sardsted. AG & Co. Kg. Germany). Urine samples were immediately tested for pH (LAQUAtwin PH-22 compact PH meter, Horiba Advanced Techno Co., Ltd, Kytoto, Japan) and specific gravity (portable salinity refractometer RF20, Extech Instruments, USA).

#### Parturition measures

At the onset of parturition, a 10-mL blood sample was collected from ewes when visible contractions were seen, or the amniotic sac was expelled, then again 4 h after the second lamb was born. Ewe blood samples were immediately measured for blood gas, chemistry, and metabolites using a blood gas analyzer (EPOC, Alere, Waltham, MA, USA). Blood samples were then processed and stored as described in the ewe prepartum measures section. Immediately after a lamb was born, a 1-mL blood sample was collected via jugular venepuncture using a 2-mL syringe and 21G 1″ needle to measure blood lactate (Lactate pro 2 LT-1730, Arkray Global Business, Inc. Japan). When a lamb was born, the time of birth, assistance at parturition (yes/no), and meconium score (1, no staining; 2, mild staining; 3, moderate staining; and 4, severe staining) were recorded (Castro-Nájera et al., 2006).

#### Postpartum measures

Four hours after birth, lambs were weighed, sexed, ear tagged, measured for body morphology (crown-rump and forelimb length, thoracic and abdominal circumference, and crown width), and rectal temperature was recorded. A 10-mL colostrum sample from the ewe was also collected. At 24 h of age, a 3-mL blood sample was collected from lambs using a 3-mL syringe and 21G 1″ needle via jugular venepuncture and dispensed into a 5-mL clot activator vacutainer tube. The sample was processed and stored as described in the ewe prepartum measures section. The remaining blood in the syringe was measured for blood glucose (Abbots Freestyle Optium Neo. Melbourne, VIC, Australia). At 24 and 72 h, both the ewe and lambs were weighed, and the ewes were body condition scored. Lamb survival was monitored weekly until 39 d postpartum at which point the lambs were weighed again.

### Retrospective biological sample analysis and parturition duration

Blood samples were analyzed for calcium, cholesterol, phosphate (reagent; Thermo Fisher), beta hydroxy butyrate, nonesterified fatty acids (**NEFAs**), and magnesium (reagent; Randox) via Konelab20Xti (Thermo Scientific, Finland) by the University of Sydney, Veterinary Pathology Diagnostic Services, NSW, Sydney. Colostrum and lamb serum were analyzed for immunoglobulin G (**IgG**) content via a previously validated radial-immunodiffusion (**RID**) assay developed at the University of Adelaide’s Veterinary Diagnostic Laboratory (Roseworthy Campus, Roseworthy, South Australia, Australia) with methods described by Brougham et al. (2020).

Parturition duration was recorded using a CCTV system within the indoor animal housing facility, and footage was backed up daily onto external hard drives. Parturition duration was determined by reviewing infrared video surveillance where the start of parturition was considered to be during the second stage of labor when the ewe is actively pushing/contracting and the end of parturition being the expulsion of the fetus. The start of parturition for the second-born lamb was considered to be the expulsion of the first-born lamb. Lamb shape and size were determined by calculating ponderal index {birth weight [kg]/[crown-rump length (m)]^3^} and body mass index {birth weight [kg]/[crown-rump length(m^2^)]}, respectively with equations provided by Baxter et al. (2008).

### Sample size estimations

Parturition duration was the outcome of interest in this study and the ewe was the experimental unit used to determine sample size. The software G*Power (version 3.1.9.7) was used to estimate the sample size. A standard deviation of 0.6 (10 min) was determined from previous studies using the same facilities with parturition duration as one of the outcomes of interest. The inputs provided to the software consisted of a standardized effect size = 0.5, α = 0.05, and power = 0.8. Based on the output from the software, the total number of ewes per treatment was estimated to be 35.

### Statistical analysis

The statistical software SPSS (IBM) version 29 was used for statistical analysis and a *P* value of ≤0.050 was considered significant and a *P* value between ≤0.100 and >0.050 was considered marginally significant. A generalized linear mixed effects model was used to analyze scale variables and a mixed effects logistic regression was used to analyze dichotomous variables. A repeated measure mixed model analysis was used to assess the effect of the dietary treatment over time on mineral and metabolite concentrations. A Kaplan–Meier parametric time failure model was used to analyze time events (parturition duration) and a Cox proportional hazards regression model was used to analyze lamb survival on the basis of satisfying the model’s assumptions. The experimental unit used was the ewe due to each animal receiving the experimental diet in individual pens. When analyzing ewe data, the dependent variables consisted of blood parameters, parturition duration and difficulty, urine pH, LW, and BCS. The fixed independent variables included treatment (positive DCAD vs. negative DCAD) and ewe age. Replicate was fitted as a random variable. When analyzing lamb data, dependent variables consisted of blood parameters, LW, meconium score, survival, body morphology, and rectal temperature. The fixed independent variables included treatment (positive DCAD vs. negative DCAD), sex, birth order, and ewe age. Replicate was fitted as a random variable. Ten ewes from the negative DCAD group and 12 ewes from the positive DCAD were removed from the trial either due to not eating the diet (negative DCAD *n *= 5 and positive DCAD *n *= 3), metabolic disease (negative DCAD *n *= 1 and positive DCAD *n *= 2), mastitis (negative DCAD *n *= 2 and positive DCAD *n *= 2), or the ewe did not give birth to twins (negative DCAD *n *= 2 and positive DCAD *n *= 5).

## Results

The duration of dietary treatment was similar for both the positive and negative DCAD groups when both replicates (early and late mated groups) were combined (*P *= 0.552); however, there was a replicate effect where negative DCAD ewes in replicate 2 received the dietary treatment for 4.7 d longer compared with the positive DCAD ewes (*P *< 0.05; Table 3). There were no differences in daily feed intake during gestation between the negative DCAD ewes (1,139.1 ± 53.2 g/d) and the positive DCAD ewes (1,210.8 ± 53.2 g/d; *P *= 0.48). The percent of ewes that ate their full ration did not differ between the positive DCAD ewes (30.4 ± 9.8 %) and negative DCAD ewes (42.3 ± 9.9 %; *P *= 0.589).

**Table 3.** The duration (days) of dietary supplementation for the positive and negative DCAD treatment groups prepartum

	Positive DCAD	Negative DCAD	*P* value
*n*	23	26	
Rep 1 and 2, days	23.0 ± 1.3	21.9 ± 1.2	0.552
*n*	16	15	
Rep 1, days	26.0 ± 1.5	22.7 ± 1.5	0.139
*n*	7	11	
Rep 2, days	16.1 ± 1.9	20.8 ± 1.7	0.049

Data presented as mean ± SEM.

### Ewe blood metabolites

There were no differences in blood metabolites between the positive and negative DCAD groups except for negative DCAD ewes having marginally significantly greater blood ketones at dG 145 compared with the positive DCAD ewes (*P *< 0.01; Table 4). There were no differences in blood metabolites pre- and postpartum measured via EPOC between the positive and negative DCAD groups (*P *> 0.05; Table 5). When analyzed using a repeated measure mixed model, there were no differences in blood metabolites between the positive and negative DCAD groups during the gestation timepoints (dG 130, 140, and 145; *P *> 0.05) nor all timepoints (dG 130, 140, 145, and pre- and postpartum; *P *> 0.05).

**Table 4.** Blood metabolite concentrations of twin-bearing ewes fed either a positive or negative DCAD diet measured on dG 130, 140, 145, and immediately before (pre-) and postpartum

	Positive DCAD	Negative DCAD	*P* value
dG 130			
* n*	22	26	
Blood glucose, mmol/L	2.8 ± 0.2	2.7 ± 0.2	0.629
Blood Ketones, mmol/L	0.9 ± 0.1	0.8 ± 0.1	0.586
Cholesterol, mmol/L	2.2 ± 0.1	2.3 ± 0.1	0.904
NEFA, mmol/L	0.8 ± 0.1	1.0 ± 0.1	0.217
BHB, mmol/L	0.8 ± 0.1	0.8 ± 0.1	0.941
dG 140			
* n*	21	26	
Blood glucose, mmol/L	3.0 ± 0.1	2.9 ± 0.1	0.647
Blood ketones, mmol/L	0.4 ± 0.1	0.7 ± 0.1	0.138
Cholesterol, mmol/L	2.2 ± 0.1	2.2 ± 0.1	0.868
NEFA, mmol/L	0.3 ± 0.1	0.5 ± 0.1	0.160
BHB, mmol/L	0.4 ± 0.1	0.6 ± 0.1	0.144
dG 145			
* n*	21	25	
Blood glucose, mmol/L	3.3 ± 0.2	3.0 ± 0.1	0.206
Blood ketones, mmol/L	0.5 ± 0.1	0.7 ± 0.1	0.099
Cholesterol, mmol/L	2.3 ± 0.1	2.2 ± 0.1	0.769
NEFA, mmol/L	0.3 ± 0.1	0.5 ± 0.1	0.133
BHB, mmol/L	0.5 ± 0.1	0.7 ± 0.1.	0.161
Prepartum			
* n*	9	18	
Blood glucose, mmol/L	4.1 ± 0.8	5.0 ± 0.5	0.334
Blood Ketones, mmol/L	1.2 ± 0.4	1.5 ± 0.3	0.545
Cholesterol, mmol/L	2.1 ± 0.1	2.2 ± 0.1	0.519
NEFA, mmol/L	1.8 ± 0.3	1.7 ± 0.2	0.724
BHB, mmol/L	1.0 ± 0.3	1.3 ± 0.2	0.415
Postpartum			
* n*	14	20	
Blood glucose, mmol/L	4.2 ± 0.5	4.4 ± 0.4	0.854
Blood ketones, mmol/L	4.2 ± 0.5	4.4 ± 0.4	0.854
Cholesterol, mmol/L	1.8 ± 0.1	2.0 ± 0.1	0.053
NEFA, mmol/L	0.7 ± 0.1	0.7 ± 0.1	0.815
BHB, mmol/L	0.5 ± 0.1	0.6 ± 0.1	0.482

Data presented as mean ± SEM.

BHB, beta-hydroxybutyrate; dG, day of gestation; NEFA, nonesterified fatty acids.

**Table 5.** Blood metabolite concentrations pre- and postpartum of twin-bearing ewes fed either a positive or negative DCAD measured via EPOC

	Positive DCAD	Negative DCAD	*P* value
Prepartum			
* n*	8	17	
Glucose, mmol/L	4.8 ± 0.8	6.1 ± 0.6	0.199
Lactate, mmol/L	2.5 ± 0.9	3.3 ± 0.6	0.406
BUN, mg/dL	19.0 ± 3.1	21.6 ± 2.2	0.504
Creatinine, mg/dL	1.2 ± 0.1	1.2 ± 0.1	0.776
Postpartum			
* n*	13	19	
Glucose, mmol/L	4.7 ± 0.6	5.4 ± 0.5	0.377
Lactate, mmol/L	2.8 ± 0.5	3.4 ± 0.4	0.406
BUN, mg/dL	20.1 ± 2.4	22.3 ± 2.0	0.614
Creatinine, mg/dL	1.5 ± 0.1	1.4 ± 0.1	0.228

Data presented as mean ± SEM.

Abbreviation: BUN, blood urea nitrogen.

### Ewe circulating minerals

Positive DCAD ewes had greater serum magnesium at dG 130 (*P *= 0.03) and greater serum phosphate concentrations at dG 145 (*P *< 0.05), compared with negative DCAD ewes (Table 6). Positive DCAD ewes had marginally significantly greater serum magnesium concentrations at dG 140 (*P *= 0.08) compared to negative DCAD ewes, however, at the start of parturition positive DCAD ewes had lower serum magnesium compared with negative DCAD ewes (*P* = 0.09). Positive DCAD ewes had marginally statistically greater serum calcium concentrations at dG 130 (*P *= 0.09) but marginally lower ionized calcium concentrations prepartum compared with the negative DCAD ewes (*P *= 0.09). When analyzed using a repeated measure mixed model, there were no differences in circulating minerals between the positive and negative DCAD groups during the gestation timepoints only (dG 130, 140, and 145; *P *> 0.050) nor all timepoints (dG 130, 140, 145, and pre- and postpartum; *P *> 0.05).

**Table 6.** Blood mineral concentrations^1^ of twin-bearing ewes fed either a positive or negative DCAD diet measured on dG 130, 140, 145 and immediately pre- and postpartum

	Positive DCAD	Negative DCAD	*P* value
dG 130			
* n*	22	26	
Calcium, mmol/L	2.27 ± 0.05	2.16 ± 0.04	0.085
Ionized calcium, mmol/L	1.11 ± 0.05	1.06 ± 0.04	0.470
Magnesium, mmol/L	1.05 ± 0.03	0.96 ± 0.03	0.025
Phosphate, mmol/L	2.02 ± 0.08	2.02 ± 0.08	0.993
dG 140			
* n*	21	26	
Calcium, mmol/L	2.30 ± 0.04	2.37 ± 0.03	0.198
Ionized calcium, mmol/L	1.11 ± 0.03	1.15 ± 0.02	0.328
Magnesium, mmol/L	1.19 ± 0.03	1.11 ± 0.03	0.081
Phosphate, mmol/L	1.82 ± 0.08	1.83 ± 0.07	0.955
dG 145			
* n*	21	25	
Calcium, mmol/L	2.46 ± 0.04	2.44 ± 0.04	0.719
Ionized calcium, mmol/L	1.09 ± 0.03	1.13 ± 0.03	0.364
Magnesium, mmol/L	1.14 ± 0.03	1.14 ± 0.03	0.952
Phosphate, mmol/L	2.14 ± 0.08	1.87 ± 0.07	0.047
Prepartum			
* n*	9	18	
Calcium, mmol/L	2.31 ± 0.06	2.36 ± 0.04	0.472
Ionized calcium, mmol/L	1.18 ± 0.08	1.35 ± 0.06	0.093
Magnesium, mmol/L	0.98 ± 0.04	1.06 ± 0.03	0.088
Phosphate, mmol/L	1.93 ± 0.17	2.03 ± 0.12	0.645
Postpartum			
* n*	14	20	
Calcium, mmol/L	2.33 ± 0.04	2.40 ± 0.04	0.238
Ionized calcium, mmol/L	1.26 ± 0.08	1.4 ± 0.07	0.202
Magnesium, mmol/L	1.09 ± 0.04	1.04 ± 0.03	0.406
Phosphate, mmol/L	2.35 ± 0.13	2.09 ± 0.11	0.144

Data presented as mean ± SEM.

^1^Ionized calcium measured in whole blood and calcium, magnesium, and phosphate were measured in serum.

Abbreviation: dG, day of gestation.

### Acid–base balance of ewes

The positive DCAD-fed ewes had a greater venous blood pH (EPOC; *P *= 0.02) and BE (ecf) prepartum (*P *= 0.04) compared with the negative DCAD-fed ewes (Table 7). The negative DCAD ewes had marginally lower venous blood chCO_3_ prepartum and blood pH (hand-held meter) postpartum compared with the positive DCAD ewes (*P *< 0.06; Table 6). Positive DCAD ewes had a greater urine pH at dG 140 (*P *= 0.08) and 145 (*P *< 0.001) and a marginally significantly greater urine pH at dG 130 compared with the negative DCAD ewes (*P *= 0.06; Table 8).

**Table 7.** Blood acid–base balance measures of twin-bearing ewes fed either a positive or negative DCAD diet

	Positive DCAD	Negative DCAD	*P* value
Blood pH (hand-held meter)			
* n*	22	25	
dG 130	7.55 ± 0.02	7.54 ± 0.02	0.642
dG 140	7.67 ± 0.03	7.71 ± 0.02	0.321
dG 145	7.64 ± 0.02	7.65 ± 0.02	0.691
* n*	8	17	
Prepartum	7.52 ± 0.06	7.59 ± 0.04	0.317
* n*	12	18	
Postpartum	7.69 ± 0.03	7.61 ± 0.02	0.060
Prepartum EPOC			
* n*	8	17	
pH	7.44 ± 0.02	7.38 ± 0.02	0.021
pCO_2_, mmHg	36.0 ± 1.9	36.4 ± 1.3	0.872
pO^2^, mmHg	41.6 ± 4.0	39.0 ± 2.7	0.589
chCO_3_, mmol/L	24.6 ± 1.3	21.5 ± 0.9	0.060
BE (ecf), mmol/L	0.5 ± 1.5	−3.6 ± 1.0	0.035
cSO_2_, %	74.2 ± 3.3	71.1 ± 2.3	0.441
Postpartum EPOC			
* n*	13	19	
pH	7.44 ± 0.02	7.41 ± 0.01	0.140
pCO_2_, mmHg	33.7 ± 1.3	36.3 ± 1.1	0.140
pO^2^, mmHg	38.7 ± 1.3	36.9 ± 1.0	0.269
chCO_3_, mmol/L	23.0 ± 0.9	23.2 ± 0.8	0.908
BE (ecf), mmol/L	−1.1 ± 1.1	−1.4 ± 0.9	0.804
cSO_2_, %	75.2 ± 2.5	70.5 ± 2.1	0.155

Data presented as mean ± SEM.

Abbreviations: dG, day of gestation; pCO_2_, partial pressure of carbon dioxide; pO^2^, partial pressure of oxygen; chCO^3^, bicarbonate; BE (ecf), base excess in extracellular fluid; cSO_2_, oxygen saturation.

**Table 8.** Urine pH and urine specific gravity of twin-bearing ewes fed either a positive or negative DCAD diet measured on dG 130, 140, and 145

	Positive DCAD	Negative DCAD	*P* value
Urine pH			
* n*	21	26	
dG 130	7.96 ± 0.17	7.50 ± 0.16	0.055
dG 140	8.10 ± 0.19	7.38 ± 0.17	0.007
dG 145	8.25 ± 0.20	7.20 ± 0.19	<0.001
Urine specific gravity			
* n*	21	26	
dG 130	1.049 ± 0.005	1.049 ± 0.005	0.952
dG 140	1.035 ± 0.004	1.030 ± 0.004	0.454
dG 145	1.032 ± 0.004	1.035 ± 0.004	0.665

Data presented as mean ± SEM.

Abbreviation: dG, day of gestation.

### Ewe body composition and parturition outcomes

There were no treatment differences in ewe LW or BCS (*P *> 0.05), except for positive DCAD ewes were marginally heavier LW 72 h postpartum compared with the negative DCAD ewes (*P *= 0.06; Table 9).

**Table 9.** LW and BCS of twin-bearing ewes fed either a positive or negative DCAD diet measured on dG 127 and 135, and 24 and 72 h postpartum

	Positive DCAD	Negative DCAD	*P* value
LW, kg			
* n*	23	26	
dG 127	87.6 ± 1.7	85.7 ± 1.6	0.422
dG 135	87.9 ± 1.7	87.5 ± 1.6	0.855
24 h postpartum	77.0 ± 1.9	74.4 ± 1.7	0.307
72 h postpartum	78.9 ± 1.7	74.5 ± 1.5	0.061
BCS			
* n*	23	26	
dG 127	3.8 ± 0.1	3.7 ± 0.1	0.343
dG 135	3.8 ± 0.1	3.6 ± 0.1	0.666
24 h postpartum	3.4 ± 0.1	3.4 ± 0.1	0.539
72 h postpartum	3.4 ± 0.1	3.3 ± 0.1	0.432

Data presented as mean ± SEM.

Abbreviation: BCS, body condition score; dG, day of gestation; LW, liveweight.

**Figure 1.**
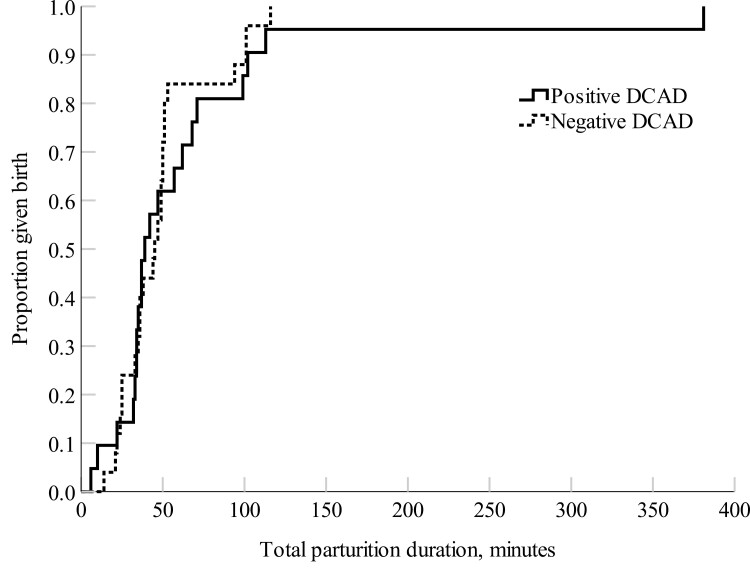
Kaplan–Meier time failure graph for the total parturition length of the positive DCAD (*n *= 21) and negative DCAD (*n *= 25) ewes. The mean total parturition duration was 99.7 ± 17.3 min for the positive DCAD ewes and 66.9 ± 5.6 min for the negative DCAD ewes, respectively.

**Figure 2.**
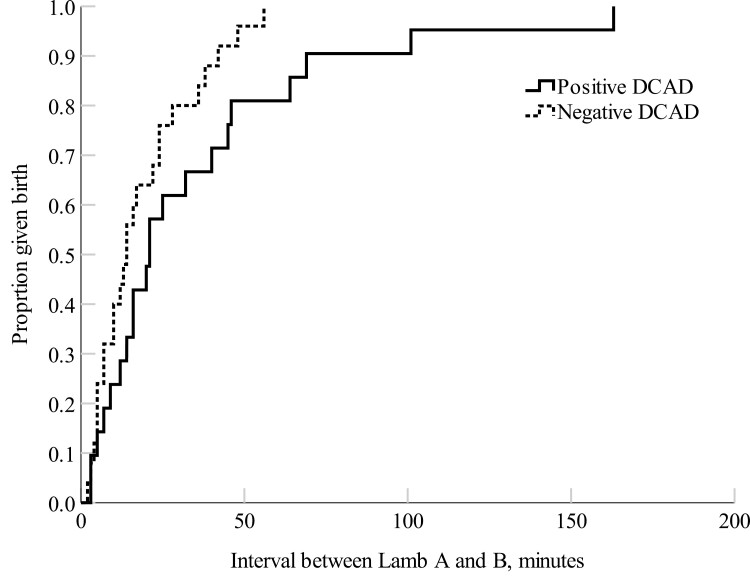
Kaplan–Meier time failure graph for the time interval between Lamb A (first-born lamb) and Lamb B (second-born lamb) being born from the positive DCAD (*n *= 21) and negative DCAD (*n *= 25) ewes. The mean parturition duration of Lamb B was 34.9 ± 8.4 min for the positive DCAD ewes and 18.5 ± 3.0 min for the negative DCAD ewes, respectively.

**Figure 3.**
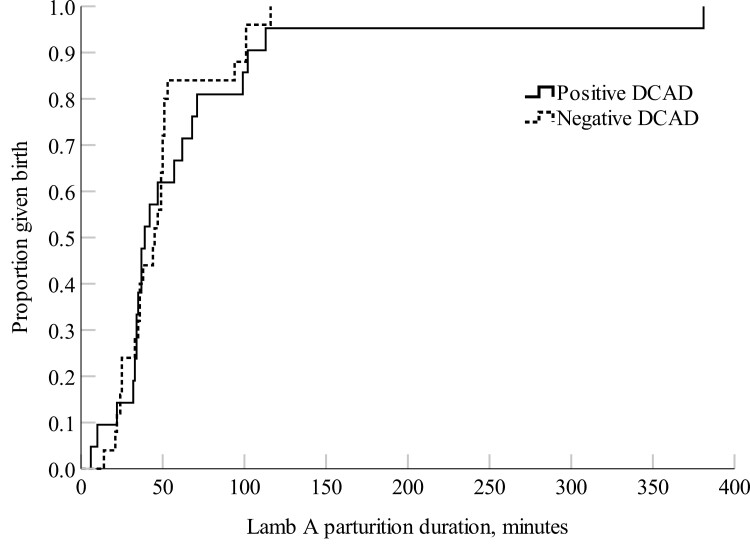
Kaplan–Meier time failure graph for the parturition duration of Lamb A (first-born lamb) of the positive DCAD (*n *= 21) and negative DCAD (*n *= 25) ewes. The mean parturition duration of Lamb A was 64.8 ± 17.0 min for the positive DCAD ewes and 48.4 ± 5.4 min for the negative DCAD ewes, respectively.

The negative DCAD ewes had a shorter total parturition duration (*P *= 0.03) and a shorter time interval between the first and second-born lambs (*P *= 0.02) compared with the positive DCAD ewes (Figures 1 and 2). There were no treatment differences in parturition duration for the first-born lamb (*P *= 0.56; Figure 3), nor the percent of ewes that required assistance at delivery (positive DCAD = 8.7 % and negative DCAD = 7.7 %; *P *= 0.90) and colostrum immunoglobulin G (positive DCAD = 36.3 ± 4.2 mg/L and negative DCAD ewes = 38.3 ± 3.6 mg/L; *P *= 0.72).

### Lamb survival, weight, and viability

**Figure 4.**
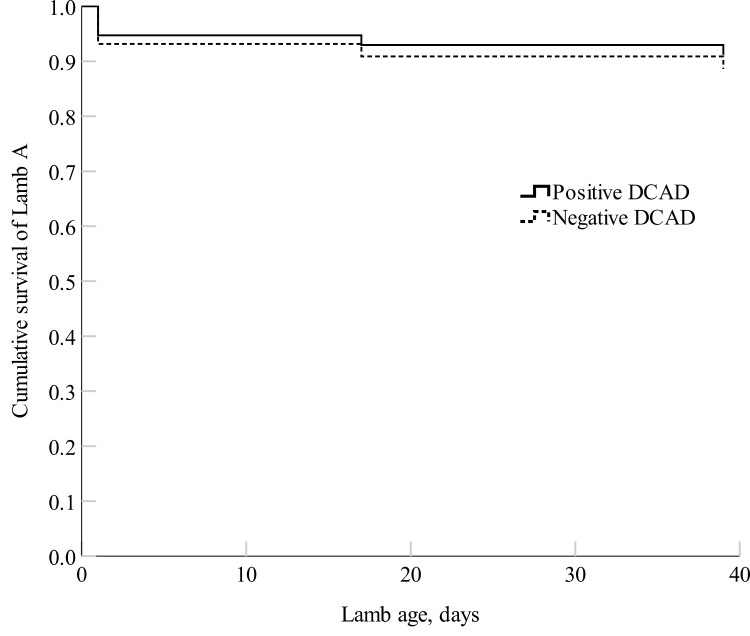
Cox proportional hazards regression model of the cumulative survival (proportion) of Lamb A (first-born lamb) from birth until day 39 (lamb marking) of age born to the positive DCAD and the negative DCAD ewes. The mean survival rate of Lamb A was 91 ± 6% (*n *= 23) for the positive DCAD group and 88 ± 8% (*n *= 26) for the negative DCAD group, respectively.

**Figure 5.**
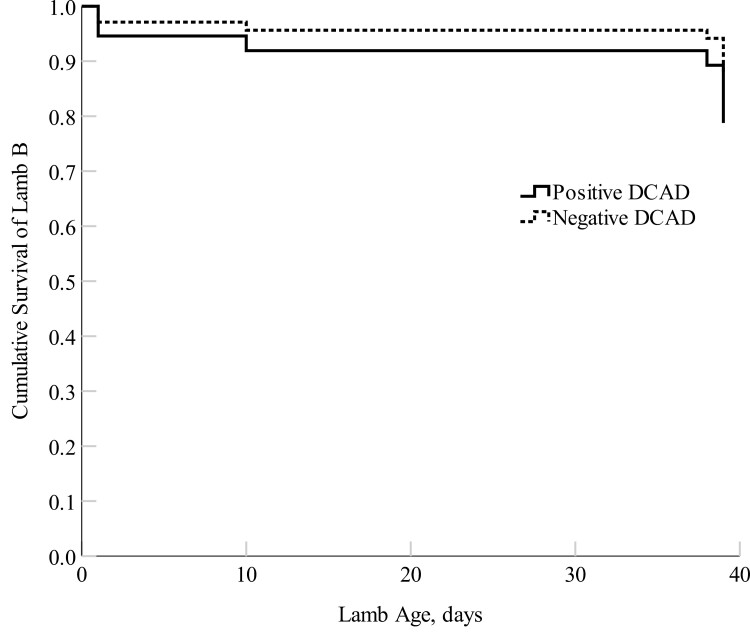
Cox proportional hazards regression model of the cumulative survival (proportion) of Lamb B (second-born lamb) from birth to day 39 (lamb marking) of age born to the positive DCAD (*n *= 23) and the negative DCAD ewes (*n *= 26). The mean survival rate of Lamb B was 78 ± 9% for the positive DCAD group and 85 ± 7% for the negative DCAD group, respectively.

There were no differences in cumulative survival for both Lamb A (first-born; *P *= 0.77) and Lamb B (second-born; *P *= 0.38) born to positive and negative DCAD-fed ewes (Figures 4 and 5). There were no differences in lamb LW at any timepoint between lambs born to positive and negative DCAD ewes (*P *> 0.05; Table 10) nor in average daily gain from 4 h to day 39 of age. There were no differences (*P *> 0.05) in lamb viability measures except for lambs born to positive DCAD ewes had a marginally greater rectal temperature at 24 h compared with lambs born to negative DCAD ewes (*P* = 0.10; Table 11). Lambs born to negative DCAD ewes had a longer shoulder-forelimb and hip-hindlimb length compared with lambs born to positive DCAD ewes (*P *= 0.03; Table 12). Lambs born to negative DCAD ewes had a marginally significantly longer crown-rump length and a smaller ponderal index compared with lambs born to positive DCAD ewes (*P *< 0.10).

**Table 10.** Liveweight of lambs at 4, 24, and 72 h and 39 d of age that were born from ewes fed either a positive or negative DCAD diet

	Positive DCAD	Negative DCAD	*P* value
*n*	45	51	
4 h, kg	4.3 ± 0.1	4.5 ± 0.1	0.176
*n*	43	50	
24 h, kg	4.4 ± 0.1	4.6 ± 0.1	0.280
72 h, kg	4.9 ± 0.1	5.1 ± 0.1	0.263
*n*	39	45	
D 39, kg	12.1 ± 0.5	12.2 ± 0.4	0.951
ADG^1^, g/d	199.0 ± 0.1	193.0 ± 0.1	0.665

Data presented as mean ± SEM.

^1^Average daily gain from 4 h to d 39 of age.

**Table 11.** Viability measures of lambs at birth, 4 and 24 h of age that were born from ewes fed either positive or negative DCAD diet

	Positive DCAD	Negative DCAD	*P* value
*n*	45	51	
Meconium staining, *n*	1.7 ± 0.1	1.9 ± 0.2	0.442
*n*	21	30	
0 h blood lactate	10.8 ± 0.9	9.6 ± 0.8	0.364
*n*	43	50	
4 h rectal temperature, °C	39.3 ± 0.1	39.3 ± 0.1	0.704
24 h rectal temperature, °C	39.4 ± 0.1	39.2 ± 0.1	0.099
24 h blood glucose, mmol/L	5.8 ± 0.2	5.7 ± 0.2	0.655
24 h serum IgG, mg/mL	25.3 ± 1.5	26.6 ± 1.4	0.529

Data presented as mean ± SEM.

Abbreviation: IgG, immunoglobulin G.

**Table 12.** Body morphology measures of lambs at 4 h of age that were born from ewes fed either a positive or negative DCAD diet

	Positive DCAD	Negative DCAD	*P* value
*n*	45	51	
Crown-rump length, cm	50.1 ± 0.6	51.8 ± 0.6	0.050
Shoulder-forelimb length, cm	40.5 ± 0.4	41.7 ± 0.4	0.033
Hip-hindlimb length, cm	43.9 ± 0.5	45.6 ± 0.4	0.010
Thoracic circumference, cm	36.5 ± 0.4	37.3 ± 0.4	0.164
Abdominal circumference, cm	34.9 ± 0.6	35.8 ± 0.5	0.268
Crown width, mm	6.3 ± 0.05	6.3 ± 0.05	0.921
Ponderal index, *n*	34.5 ± 1.0	32.0 ± 0.9	0.074
Body mass index, *n*	17.0 ± 0.4	16.5 ± 0.4	0.313

Data presented as mean ± SEM.

## Discussion

The objective of our study was to determine whether feeding a negative DCAD diet would improve ewe metabolic state and mineral status, and in return, decrease parturition length and improve lamb viability. The negative DCAD ewes had marginally greater ionized calcium and serum magnesium concentrations prepartum (but not at any other timepoint) and a shorter total parturition duration compared with the positive DCAD ewes which partially supports the hypothesis of this study. Limitations of the study include relatively low numbers of ewes and lambs to evaluate dichotomous outcomes including survival, birth difficulty, and disease. Further, blood sampling close to parturition may have disrupted normal parturition behavior, as a human intervention during the birth process can be a risk factor for dystocia (Jacobson et al., 2020). A further limitation is that, while urine pH was lower for negative DCAD-fed ewes, the urine pH values did not reach the levels required for creating a mild metabolic acidosis (urine pH < 6.8; Horst et al., 1997; Goff, 2008). This indicates that despite differences in diet design and analysis, the dietary manipulation was not fully effective in creating metabolic acidosis. These limitations suggest that the increase in ionized calcium and serum magnesium seen in the negative DCAD ewes was due to other factors and not dietary acidification. One factor that could have influenced this is the difference in the form of supplemented magnesium with the negative DCAD receiving the more available magnesium sulfate and the positive DCAD magnesium oxide.

Calcium is an important mineral involved in smooth muscle contractions, including uterine contractions during parturition. Weak uterine contractions during parturition are known as uterine inertia, a common form of dystocia associated with dairy cows experiencing hypocalcemia and subclinical hypocalcemia (Curtis et al., 1983; Goff, 2008). Feeding dairy cows a negative DCAD diet in late gestation can shift the acid–base balance to a mild metabolic acidosis (Gaynor et al., 1989). This shift increases the small intestine, bone, and kidney cells' responsiveness to parathyroid hormone resulting in an increase in either or both total calcium and ionized calcium concentrations (Oetzel et al., 1991; Phillippo et al., 1994), particularly through calcium mobilization from bones (Block, 1984; Gaynor et al., 1989; Phillippo et al., 1994). Studies in sheep have shown that feeding a negative DCAD diet improves the calcium status by achieving a mild metabolic acidosis (Espino et al., 2003; Las et al., 2007; Wilkens et al., 2016; Freitag et al., 2021). Therefore, it was expected in our study that ewes receiving a negative DCAD group would have greater circulating calcium concentrations; however, no improvements in serum calcium were seen, except for negative DCAD ewes having a marginally greater ionized calcium prepartum. However, it is important to note that the positive DCAD ewes had half the sample size at this timepoint due to it being a time-sensitive measure and the values were within normal ranges in sheep (Radostits et al., 2006; Munn et al., 2024). The positive DCAD group had a greater concentration of serum magnesium and a marginally greater concentration of serum calcium prediet transition, which may have limited the effectiveness of the negative DCAD diet. Furthermore, calcium intake may affect the effectiveness of a negative DCAD diet in increasing calcium concentrations, making it difficult to compare results from literature with different concentrations of calcium, DCAD and feeding periods (Liesegang, 2008). Lastly, the ewes in the negative DCAD group were not sufficiently acidified in the current study to elicit a difference in calcium concentrations, despite the diet being formulated to achieve a mild metabolic acidosis.

As previously mentioned, the aim of a negative DCAD diet is to shift the acid–base balance of the animal to a mild metabolic acidosis to increase calcium concentrations. The negative DCAD ewes had a significantly lower urine pH at both dG 140 and 145, lower blood pH via EPOC, blood base excess, and a marginally significantly lower blood bicarbonate concentrations prepartum compared with the positive DCAD group, which agrees with other studies (Espino et al., 2003; Las et al., 2007; Liesegang, 2008; Wilkens et al., 2016; Freitag et al., 2021). However, it is important to note that the positive DCAD diet was formulated to have a high DCAD by using sodium bicarbonate, which would explain the great difference in blood base excess and blood bicarbonate. Despite this, the negative DCAD ewes in the current study had a urine pH of 7.2, whereas a urinary pH between 5.5 and 6.2 and 6.2 to 6.8 was considered effective in preventing hypocalcemia and subclinical hypocalcemia, respectively, when administering anions to dairy cows (Horst et al., 1997; Goff, 2008). However, the meta-analyses of Lean et al. (2019) and Santos et al. (2019) in cattle found that responses in blood calcium were associated with a linear reduction in DCAD, not a response to a negative DCAD. Other sheep studies conducted indoors and fed a negative DCAD diet that had an increase in calcium concentrations had a urine pH of 6.09 (Las et al., 2007) and 5.51 to 6.84 (Freitag et al., 2021). Considering <50% of ewes in both the positive and negative DCAD groups consumed the whole TMR, it may be possible that the ewes selectively ate their diet to exclude the anionic component of the diet (both dietary groups received the anionic protein meal Bio-Chlor at a rate of <2.3% AF per day). If a negative DCAD supplement/diet causes metabolic acidosis or decreased palatability caused by the addition of anions, a reduction in feed intake may occur (Lean et al., 2019; Santos et al., 2019). Therefore, it is plausible that not enough acidification occurred, as reflected in the urinary pH results, in the negative DCAD group due to majority of the ewes not eating the full ration. This creates a further limitation to the study whereby the sample size to include only ewes that ate their full ration was insufficient to draw conclusions (*n *= 5 and *n *= 7 for positive and negative DCAD ewes, respectively). Future studies using a loose mix diet may want to consider adding a binding agent, such as oil or molasses, to hold the loose mix together and enhance flavor, as this may reduce the issue in our study of ewes selectively eating the diet.

In dairy cows, low calcium status can be a risk factor for metabolic disease (Goff, 2008). A study in sheep found that hypocalcemia can further facilitate the onset of pregnancy toxemia (severe ketosis) by reducing endogenous glucose production (Schlumbohm and Harmeyer, 2003). In addition to negative DCAD diets improving calcium status, they may also provide benefits to metabolic responses. The skeletal system plays a crucial role in energy metabolism (Lee et al., 2007). Osteocalcin is a hormone derived from bone, that can upregulate glucose and fat metabolism during phases of high metabolic demand, such as lactation (Lean et al., 2014). Acidogenic diets prepartum in dairy cattle stimulate bone turnover and improve concentrations of osteocalcin, which may then lead to enhanced effects on metabolism (Rodney et al., 2018). Other studies using sheep housed in pens found that feeding a negative DCAD diet did not affect feed intake (Espino et al., 2003; Las et al., 2007); however, in the study by Robertson et al. (2022), not all flocks consumed the targeted level of supplement under grazing conditions, indicating that a negative DCAD supplement may not be palatable or appealing to sheep grazing pasture. Given that there were no significant effects on ewe feed intake, LW, or BCS, ewes did not have calcium concentrations indicative of hypocalcemia and no improvement of calcium status was seen, it is not unexpected that the negative DCAD diet did not significantly influence metabolism, especially since the necessary acidification did not occur. Further research, especially with a negative DCAD diet containing sufficient anions to shift the acid–base balance to a mild acidosis or using ewes that are low in calcium, is required to determine if a negative DCAD diet affects the metabolic state in sheep. Despite the negative DCAD ewes not being adequately acidified through dietary manipulation, a marginally statistically greater ionized calcium and magnesium was seen at the start of parturition, as well as having a shorter parturition duration.

The imbalanced supply of minerals and positive DCAD of southern Australian pastures (DACD ranging from 0 to 760 mEq/kg DM; Roche et al., 2000; Hocking Edwards et al., 2018; Robertson et al., 2022) and Australian vegetative crops and pastures may be causing a significant number of grazing sheep to be subclinically deficient in calcium and magnesium (Hocking Edwards et al., 2018; Masters et al., 2018a). Calcium and magnesium are essential minerals for the birthing process as they are involved in the production of energy for myometrial contractions, which in turn, can affect parturition length (Wray and Arrowsmith, 2012). The study by Silva and Noakes (1984) reported that experimentally inducing hypocalcemia by an intravenous infusion of ethylene-diamine tetra acetic acid, and disodium salt during parturition reduced uterine activity of ewes when plasma calcium concentrations reached below 1.0 mmol/L. In addition, the study by Ataollahi et al. (2021) demonstrated that supplementing twin-bearing ewes with magnesium and calcium in late gestation marginally statistically reduced the parturition duration and contraction length of the second-born lamb. This indicates that increased calcium and magnesium concentrations may reduce parturition length in twin-bearing ewes. In our study, the marginally significantly increased ionized calcium and serum magnesium may explain why the decrease in parturition length of the second-born lamb was detected in the negative DCAD ewes; however, as previously mentioned, it is important to note that the negative DCAD ewes had double the sample size at this timepoint. In addition, although ionized calcium is more relevant compared with total calcium when assessing calcium status (Ott et al., 2021), no differences in serum calcium were seen. To the best of our knowledge, this is the first report of the effects of a negative DCAD diet on the parturition process in sheep; however, as previously mentioned, the ewes were not adequately acidified which indicates that the increase in ionized calcium and serum magnesium may have been due to the form of magnesium in these supplements and not from a reduction in DCAD (Lean et al., 2006; Irvine, 2009). Despite the negative DCAD ewes having a short parturition duration (<90 min) and the positive DCAD ewes having a long parturition duration (>90 min; Flinn et al., 2020), there were no differences in lamb viability measures at birth, whereby meconium stain score (an indicator of oxygen deprivation from a long and/or stressful birth; Castro-Nájera et al., 2006) and lamb lactate at birth did not differ, as well as lamb survival.

As previously mentioned, calcium deficiencies may lead to dystocia in sheep (Friend et al., 2020). If dystocia does not lead directly to lamb death, the effects of parturition-induced hypoxia can also lead to stillbirths (death during or shortly after parturition) and birth injury (death within 6 d of birth; Dutra et al., 2007). In our study, a negative DCAD diet did not affect lamb survival at birth or any other timepoint. Although the conclusion of a negative DCAD diet having no effect on lamb survival is limited by the sample size in our study, as it was not designed to detect differences in survival since >450 ewes per treatment would be required to detect a difference in mortality between 5% and 10%. Despite this, the cases of dystocia in our study were caused by fetal malpresentation, which can occur regardless of ewe health (Jacobson et al., 2020). Additionally, ewes were being housed indoors which protects lambs from hypothermia and predation, and can also increase ewe-lamb bonds which can increase lamb survival rates (Stafford and Gregory, 2008), as reflected by the high lamb survival rates in our study. To the best of our knowledge, only one additional study has reported the effects of a negative DCAD diet on lamb survival rates, whereby the study by Robertson et al. (2022) found that a negative DCAD supplement did not improve lamb survival under grazing conditions; however, variation in maternal supplement intake may have limited any benefit to lamb survival. This indicates that the reduction in parturition length seen in our study by the negative DCAD diet may be more beneficial to flocks affected by subclinical hypocalcemia-related dystocia, as long as the diet is highly palatable under grazing conditions.

Maternal supplementation of calcium and magnesium in twin-bearing ewes has been shown to increase lamb viability by increasing lamb growth rates up to 4 wk of age and improving lamb immunity through increasing leukocyte oxidative burst response and antioxidant capacity (Ataollahi et al., 2018, 2020). We found no significant differences in lamb weight, growth rates, body mass index, or ponderal index; however, the negative DCAD lambs had longer front and hind limbs compared with the positive DCAD lambs. Fetal skeletal growth and mineralization are dependent on placental supply from maternal circulation (Kovacs, 2014). With the lambs born to negative DCAD ewes having longer limbs, it may be that the maternal supply of calcium and magnesium was being partitioned to fetal long bone development. This may explain why a marginally statistically greater ionized calcium and magnesium was only detected at the prepartum timepoint in the negative DCAD ewes and not during the late gestation timepoints due to the fetal skeletal mineralization; however, further studies are required to support this. The lack of differences in lamb liveweight and growth may be largely attributed to the length of maternal supplementation. In our study, maternal supplementation ceased 3 d postpartum whereas in the Ataollahi et al. (2020) study, maternal supplementation continued until 4 wk postpartum. This may also explain why immunoglobulin concentrations were not affected by diet in our study, as serum IgG in the lambs was similar between dietary groups. The other lamb viability indicators of rectal temperature, and indicator of the thermoregulatory ability of the lamb (Dwyer and Morgan, 2006), and blood glucose measured in our study were also not different between DCAD groups. To the best of our knowledge, we are the first to report the effects of a negative DCAD diet on the lamb viability; however, further research is warranted to draw stronger conclusions.

## Conclusion

To the best of our knowledge, this study is the first to describe the effects of feeding ewes a negative DCAD diet in late pregnancy on parturition duration and subsequent lamb viability. The negative DCAD diet reduced the interval between the first and second-born lamb, most likely due to the marginally greater ionized calcium and magnesium concentrations. Despite this improvement, our study is limited by the negative DCAD ewes not achieving sufficient metabolic acidification most likely due to selectively not eating the anionic component of the diet. Other studies have previously shown that a negative DCAD diet can increase the calcium status of sheep. Although the negative DCAD diet decreased urine pH in ewes, the urine pH was not low enough to reach the pH recommended for anionic diets. No differences in lamb survival were detected between the positive and negative DCAD groups; however, our sample size was not designed to sufficiently detect changes in survival rates. Further research testing a negative DCAD diet that can achieve the target urine pH is required to determine whether this diet can decrease parturition duration and improve lamb viability.

## Abbreviations

ADF: Acid detergent fiber
ADG: average daily gain
AF: as fed
BCS: body condition score
BE (ecf): base excess in extracellular fluid
BHB: beta-hydroxybutyrate
BUN: blood urea nitrogen
chCO3: bicarbonate
CP: crude protein
chCO_3_: bicarbonate
cSO_2_: oxygen saturation
DCAD: dietary cation and anion difference
dG: day of gestation
DM: dry matter
G: gauge
IgG: immunoglobulin g
LW: live weight
ME: metabolizable energy
NDF: neutral detergent fiber
NEFA: nonesterified fatty acid
pCO_2_: partial pressure of carbon dioxide
pO^2^: partial pressure of oxygen
TMR: total mixed ration
